# Hypoxia-induced epigenetic regulation of breast cancer progression and the tumour microenvironment

**DOI:** 10.3389/fcell.2024.1421629

**Published:** 2024-08-30

**Authors:** Alina L. Capatina, Jodie R. Malcolm, Jack Stenning, Rachael L. Moore, Katherine S. Bridge, William J. Brackenbury, Andrew N. Holding

**Affiliations:** ^1^ Department of Biology, University of York, York, United Kingdom; ^2^ York Biomedical Research Institute, University of York, York, United Kingdom

**Keywords:** breast cancer, hypoxia, microenvironment, oestrogen receptor, triple negative breast cancer, epigenetics

## Abstract

The events that control breast cancer progression and metastasis are complex and intertwined. Hypoxia plays a key role both in oncogenic transformation and in fueling the metastatic potential of breast cancer cells. Here we review the impact of hypoxia on epigenetic regulation of breast cancer, by interfering with multiple aspects of the tumour microenvironment. The co-dependent relationship between oxygen depletion and metabolic shift to aerobic glycolysis impacts on a range of enzymes and metabolites available in the cell, promoting posttranslational modifications of histones and chromatin, and changing the gene expression landscape to facilitate tumour development. Hormone signalling, particularly through ERα, is also tightly regulated by hypoxic exposure, with HIF-1α expression being a prognostic marker for therapeutic resistance in ER^+^ breast cancers. This highlights the strong need to understand the hypoxia-endocrine signalling axis and exploit it as a therapeutic target. Furthermore, hypoxia has been shown to enhance metastasis in TNBC cells, as well as promoting resistance to taxanes, radiotherapy and even immunotherapy through microRNA regulation and changes in histone packaging. Finally, several other mediators of the hypoxic response are discussed. We highlight a link between ionic dysregulation and hypoxia signalling, indicating a potential connection between HIF-1α and tumoural Na^+^ accumulation which would be worth further exploration; we present the role of Ca^2+^ in mediating hypoxic adaptation via chromatin remodelling, transcription factor recruitment and changes in signalling pathways; and we briefly summarise some of the findings regarding vesicle secretion and paracrine induced epigenetic reprogramming upon hypoxic exposure in breast cancer. By summarising these observations, this article highlights the heterogeneity of breast cancers, presenting a series of pathways with potential for therapeutic applications.

## Graphical Abstract

**Figure:**
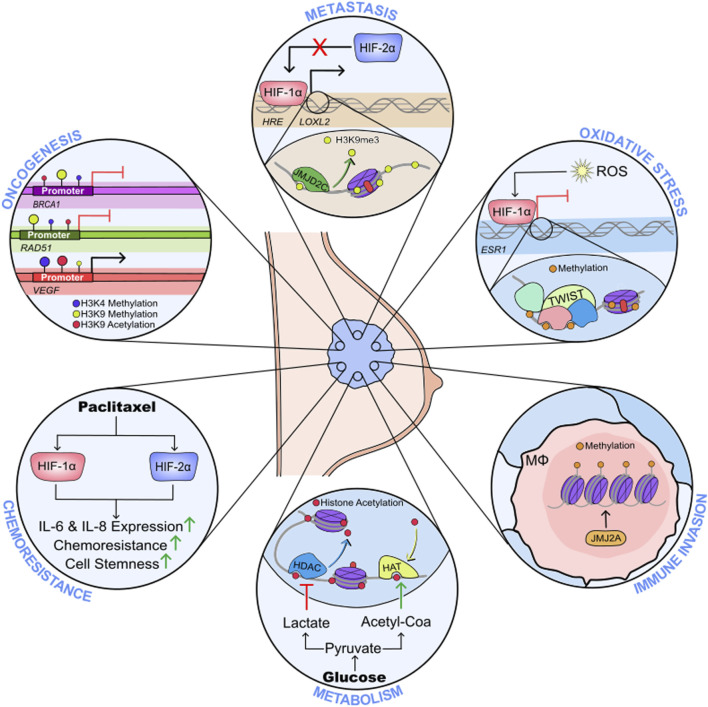
Hypoxia-mediated epigenetic regulation of breast cancer progression.

## Introduction

Tumour biology is underpinned by a complex set of interconnected signalling pathways, cellular interactions, and regulatory mechanisms that feed into the process of tumorigenesis and progression. There is much interest in understanding the nuances of these processes, as they offer valuable insights into the therapeutic vulnerabilities of breast cancer. One area of success is in the generation of information from next generation-sequencing and mass spectrometry-based proteomics from large cohorts of patients (e.g., TCGA, Metabric etc.) (Curtis et al., 2012; Dawson et al., 2013; Pereira et al., 2016; Cancer Genome Atlas Research Network et al., 2013; Ciriello et al., 2015; Cancer Genome Atlas Network, 2012; Liu J. et al., 2018) which has enabled us to profile the transcriptional and translational adaptations that malignant cells undergo to survive and proliferate. However, to understand how these adaptations occur it is critical to investigate the physiological processes that precede them, such as epigenetic modification.

Indeed, studies have identified epigenetic markers that could help profile the severity of breast tumours (Bao et al., 2019; Sarvari et al., 2022; Zhang W. et al., 2022). One such study highlights the important role of epigenetics in tumour progression, investigating 880 patients suffering from invasive breast cancer, found high levels of H3K18, H3K9, H4K12, H4K16 acetylation and H4R3, H3K4 and H4K20 methylation were inversely correlated with tumour grade. Other epigenetic modifications, for example loss of H4K16 acetylation, were identified as markers of malignancy, while decreased methylation of H4R3 and acetylation of H3K9 and 16 were correlated with increased tumour size (Elsheikh et al., 2009). Associations between histone modification patterns and breast cancer subtypes, resistance to therapy or metastases have been found in many other studies (Grosselin et al., 2019; Xi et al., 2018; Hirukawa et al., 2018; Rifaï et al., 2018; Zhuang et al., 2020; Bhan et al., 2013). In fact, metastatic behaviour (high H4R3me2 and H3K9ac, associated with lymph node metastasis), breast cancer subtype (H3K4 acetylation or methylation differentiates between breast cancer subtypes), and even progression stage (early and late stages: H3K4ac high, late stages: H3K4me3 high) have their own epigenetic signature (Elsheikh et al., 2009; Messier et al., 2016). Equally, the epithelial-mesenchymal transition (EMT) is associated with H3K27ac and loss of H3K27me3, has been shown to correlate strongly with better disease prognosis (Yoo and Hennighausen, 2012; Thakur et al., 2022). These literature observations support the major role of epigenetic modifications in breast cancer progression and highlight the value of a better understanding of their cellular regulation.

Since the discovery of the Hypoxia-Inducible Factors (HIFs) (Wang and Semenza, 1995), there has been significant interest in the interplay of these proteins and cancer progression. The level of oxygenation of tissue in breast and solid tumours is a key feature of the microenvironment, and tumours typical exhibit a shortage of oxygen (hypoxia), with oxygen partial pressure (PO_2_) ranging from 2.5 to 28 mm of mercury (Hg), compared to a PO_2_ of 65 mmHg in normal breast tissue (Vaupel et al., 2007). Triple negative breast cancer (TNBC) tumours particularly suffer from hypoxia due to uncontrolled cell proliferation outpacing vascularisation, resulting in insufficient oxygen delivery to the tumour (Srivastava et al., 2023). Therefore, aberrant proliferation leads to an oxygen partial pressure (pO_2_) gradient within the tumour, altering the transcriptional programs of cells throughout, and creating heterogeneity within tumours (Helmlinger et al., 1997; Lan et al., 2018; Bhise et al., 2023). The impact of hypoxia on breast cancer is significant, studies have shown that activation of HIFs leads to worse outcomes, tumour progression and significant increase in metastasis (Jögi et al., 2019; Cao et al., 2023; Wicks and Semenza, 2022; Schito et al., 2012; Wong et al., 2011; Bos et al., 2001). Beyond HIFs, several demethylases have been identified as oxygen dependent (Chakraborty et al., 2019; Batie et al., 2019; Qian et al., 2019; Liao and Zhang, 2020), the interplay between chromatin state and hypoxia is therefore broad acting and plays a significant role in the epigenetic state of the cell.

## Hypoxia and the molecular characteristics of the breast cancer microenvironment

### Hypoxia and metabolism

Cancer cell expansion and proliferation are sustained by a range of physiological changes including alterations in their energy metabolism and adaptation to low oxygen availability. Therefore, a better understanding of the metabolic shifts undertaken by breast cancer cells in the context of hypoxia is required in order to understand how glycolytic, tricarboxylic acid (TCA) cycle, mitochondrial electron transport chain and amino acid metabolic changes can shape the tumour genetic and epigenetic landscape, promoting malignancy.

Rapid proliferation calls for rapid energy production; this is why often in cancer, glucose becomes the main energetic fuel (Zheng, 2012). This phenomenon is known as the Warburg effect and is one of the metabolic hallmarks of cancer (Schwartz et al., 2017; Vander Heiden et al., 2009). A range of molecular adaptations are needed to sustain such a metabolic shift. Breast cancer cells require increased import of glucose to fuel their energetic needs. This demand is met by upregulating the glucose transporter protein 1 (GLUT1) (Oh et al., 2017; Hussein et al., 2011). In oestrogen receptor positive (ER^+^) breast cancer, GLUT1-dependent glucose uptake is regulated by oestrogen stimulation; thus, ER activation upregulates GLUT1 mRNA expression and protein levels, as well as functionally regulating the activity of GLUT1 and 4 via Akt activation (Medina et al., 2003; Sun et al., 2014). Furthermore, ER activation can dictate the impact of glucose availability on metabolic regulation. As such, when glucose is abundant, oestrogen will promote glycolytic metabolism inhibiting the activity of pyruvate dehydrogenase (PDH) and thus suppressing the conversion of pyruvate to acetyl CoA and its subsequent entry into the tricarboxylic acid cycle; conversely, under glucose deprivation, oestrogen will enhance oxidative phosphorylation, via PDH activation (O’Mahony et al., 2012). This modulation of PDH activity and subsequent metabolic reference has been shown to occur via oestrogen interaction with ERα and ERβ, potentially via AMPK signalling (O’Mahony et al., 2012). In hypoxia, ERα-mediated glycolysis is regulated also by HIF-1α, and the two together shape the epigenetic landscape in breast cancer cells by altering the activity of histone demethylases, such as jumonji-C domain–containing protein 2B (JMJD2B), or impacting cell cycle progression (Thakur et al., 2022; Yang et al., 2010).

In cancer, intracellular lactate accumulation is often observed, potentially due to a shift in the expression of lactate shuttle molecules, especially, through an upregulation of the Monocarboxylate transporter (MCP) system (Li et al., 2022a). Typically, MCP-1 (lactate importer) is more commonly expressed under physiological conditions, while cancer cells upregulate the MCP-4 (lactate exporter), as a consequence of HIF1-α activation, to overcome intracellular dysregulations triggered by a build-up of lactate (Li et al., 2022a; Parks et al., 2011; Halestrap, 2013), (Lock et al., 2013; Becker, 2020; Ratner, 1990; Chouaib et al., 2017; Chen et al., 2023), (Li et al., 2022a; Parks et al., 2011; Halestrap, 2013).

The increase in intracellular lactate associated with cancer facilitates its functions as a histone deacetylase (HDAC) inhibitor, promoting histone hyperacetylation, and thus altering the transcriptional activity in the tumour cells. Lactate has been associated with elevated H4 acetylation, which is thought to enhance breast cancer progression and contribute to a more detrimental outcome (Latham et al., 2012; San-Millán et al., 2019; Martinez-Outschoorn et al., 2011). Increased glycolytic activity was also shown to enhance histone lactoylation, in particular H3K18; this mechanism has been associated with enhanced expression of the c-Myc oncogene. C-Myc then promotes lactate-dependent expression of serine/arginine-rich splicing factor 10 (SRSF10), which induces alternative splicing of genes such as the apoptosis inhibitor, Bcl-x, and enhances tumour survival (Pandkar et al., 2023). Furthermore, in breast cancer patients, lactate promotes demethylation of HIF-1α and facilitates tumour progression even in the absence of hypoxia, while in prostate cancer lactate was shown to promote angiogenesis through direct lactoylation of HIF-1α (Becker et al., 2020; Coronel-Hernández et al., 2021; Gong et al., 2024).

The TCA cycle also plays a key role in breast cancer progression. Acetyl CoA is a key metabolite of the TCA cycle, and is also the main donor of acetyl groups for post-translational modifications, thus regulating histone acetylation and chromatin packaging. Different patterns of histone acetylation have been associated with different types of breast cancer. In ER^+^ lines (MCF7), an increase in H3K4 acetylation at promoters regulating ER-dependent endocrine responses was observed, as opposed to non-tumorigenic normal-like breast cell lines lines (MCF10A) which exhibited increased H3K4 acetylation at promoters regulating cell adhesion, e.g., E-cadherin. In triple negative lines (MDA-MB-231s) an upregulation in genes controlling proliferation or cell cycle progression in response to serum availability was associated with enhanced histone acetylation (Messier et al., 2016; Subik et al., 2010). Interestingly, under extensive exposure to hypoxia, ER^+^, ER^−^ HER2^+^ and triple negative lines seem to downregulate the Pyruvate Dehydrogenase E1β (PDHE1β) subunit, which is part of the PDH complex, otherwise responsible for acetyl CoA synthesis. This downregulation is believed to promote glycolytic metabolism, facilitating the Warburg effect (Yonashiro et al., 2018). Conversely, other studies suggest that HER2^+^ breast cancer lines that carry the PI3KCA mutation, leading to an overactive mTOR complex, show increased phosphorylation of the acetyl CoA synthetic enzyme, ATP citrate lyase (ACYL), via mTORC2 phosphorylation. Hyperphosphorylated ACYL then supports tumour growth and mitochondrial hyperpolarisation (change in membrane potential due to reduced activity of mitochondrial complex I, thus low oxidative phosphorylation (OXPHOS) rates) in an acetyl CoA-dependent manner, results which were not recapitulated in triple negative lines (Chen et al., 2016; Forkink et al., 2014). Furthermore, acetyl CoA plays a crucial role in the survival of cancer stem cells through the process of lipogenesis which is essential for cancer cell transformation (Menendez and Lupu, 2007), with a general trend indicating that acetylation events play key roles in early stages of breast cancer, while methylation appears to be more important in the later/metastatic stages (Messier et al., 2016).

The metabolic preference of the tumour for aerobic glycolysis leads to downregulation of TCA enzymes such as Isocitrate dehydrogenase 1 (IDH1) (Islam et al., 2017). A study looking at tissue expression of IDH1 in patients carrying invasive ductal carcinoma showed that IDH1 levels decreased as the cancer progressed. Furthermore, IDH1 expression seemed to be correlated with HER2 expression, while ER and PR levels appeared to have no impact on the enzyme levels. Furthermore, the same study proposes a mechanism to explain the regulation of IDH1 expression in TNBC cell lines, by showing that two micro RNAs, miR-32-5p and miR-92b-3p interfere with the transcription of IDH1. Low IDH1 levels seem to increase migration potential in TNBC cell lines, while ER^+^ lines responded to IDH1 depletion both by increased invasive capacity and elevated proliferation (Liu W-S. et al., 2018). These observations highlight the essential role of TCA enzymes in breast cancer progression, but also unravel the heterogeneous regulatory mechanisms associated with different subtypes. Metabolically, IDH1 converts isocitrate to α-ketoglutarate, however, mutations of IDH1/2 are frequent in cancer, and lead to conversion of α-ketoglutarate to 2-hydroxyglutarate (2-HG) (Liu et al., 2023). The accumulation of 2-HG and the depletion of α-ketoglutarate act as a promoter of hypoxia, by freeing HIF-1α from the oxygen-sensing prolyl hydroxylase domain enzymes (PHDs) (Zhao et al., 2009; Zhang C. et al., 2013). Interestingly, 2-HG has been shown to inhibit histone demethylation, especially at the H3K9 location, via inhibition of the jumonji-C domain–containing protein 2A (JMJ2A), a histone demethylase otherwise dependent on α-ketoglutarate (Lu et al., 2012), while promoting methylation of DNA CpG islands in gliomas (Turcan et al., 2012). Although IDH1/2 mutations are relatively rare in breast cancer they are associated with hormone receptor positive subtypes (Murugan and Alzahrani, 2022; Fathi et al., 2014).

Non-TCA metabolic enzymes have also been identified as regulators of breast cancer pathogenesis. As such, glutathione peroxidase 8 (GPX8) is an enzyme located in the endoplasmic reticulum and is responsible for regulating the redox balance within the cell (Ren et al., 2022). In TNBC, depletion of GPX8 has been shown to reduce EMT, induce loss of cancer stemness and decreased tumour growth, observations which were explained through a reduction in the interleukin 6 (IL-6) signalling via the Janus Kinase (JAK)/Signal Transducers and Activators of Transcription 3 (STAT3) pathway, with IL-6 being a known initiator of EMT (Khatib et al., 2020; Bharti et al., 2016). Oxidative stress has also been shown to promote epigenetic reprogramming i by activating a positive feedback signalling loop via miR526b/miR655, which upregulates markers of breast cancer progression (e.g., Thioredoxin Reductase 1) in otherwise poorly metastatic MCF7 cells (Shin et al., 2019).

Metabolite receptors can also contribute to pro-oncogenic epigenetic changes. The aryl hydrocarbon receptor (AHR) is constitutively active in advanced breast cancer tumours (Yang X. et al., 2008). AhR functions as a soluble receptor that is translocated to the nucleus by binding to the AhR nuclear translocator, also known as HIF-1β (Larigot et al., 2018). Interestingly, under hypoxic conditions, AhR signalling is impaired due to HIF-1α activation and subsequent hijacking of HIF-1β (Zhang M. et al., 2022). The receptor is known to bind tryptophan metabolites (kynurenines), as well as xenobiotics (Larigot et al., 2018; Kaiser et al., 2020; Heath-Pagliuso et al., 1998). AhR has been linked to *BRCA1* gene regulation, and the specific epigenetic changes associated with this receptor include hypermethylation of CpG islands, increased three-methylation and deacetylation of H3K9, increased levels of DNA methyltransferases 1, 3a and 3b, as well as higher levels of methyl binding protein 2 (Thakur et al., 2022; Hockings et al., 2006; Papoutsis et al., 2012). Furthermore, AhR is a master regulator of a range of physiological functions including mitochondrial respiration, via P450 gene expression regulation, and regulation of the hypoxia-induced N-Myc Downstream Regulated 1 protein (NDRG1), so that knockdown of the AhR, under hypoxia-like conditions, downregulates NDRG1 induction (Yang X. et al., 2008; Li et al., 2016). Conversely, AhR overexpression in ER^+^ lines, enhanced NDRG1 mediated proliferation and motility, highlighting the key role of AhR in adaptation to hypoxia (Li et al., 2016). Interestingly, NDRG1 expression in breast cancer patients seems to correlate with lymph node status and with HER2 expression, but not with hormone receptor levels, being a marker of aggressive breast cancer (Kotepui et al., 2023).

**FIGURE 1:**
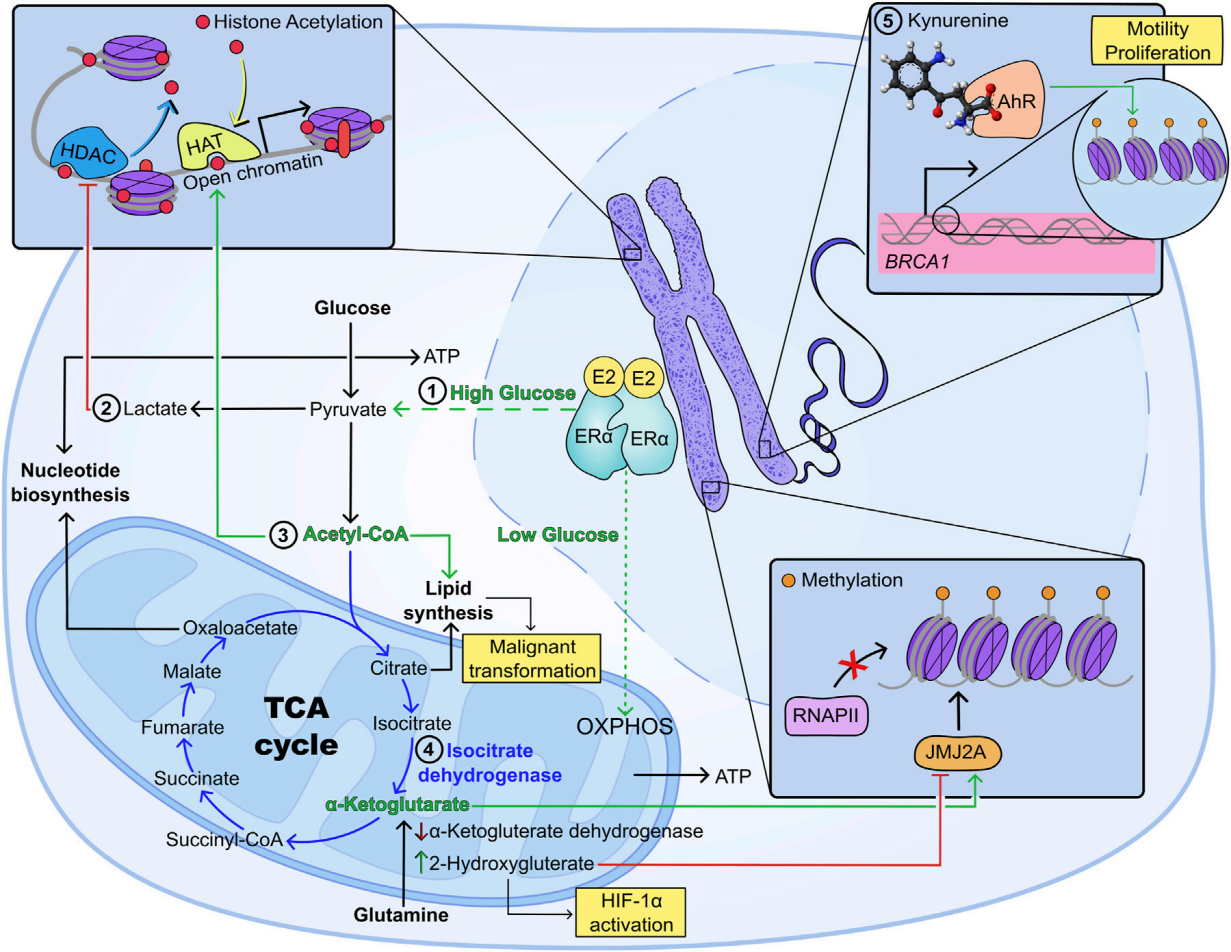
Metabolism and epigenetic regulation of breast cancer progression under hypoxia. 1) Glucose availability dictates ERα-dependent metabolic regulation, as such that high glucose promotes an increase in glycolysis, while low glucose induces preferential use of oxidative metabolism (O’Mahony et al., 2012); 2) increased aerobic glycolysis causes intracellular buildup of lactate which inhibits histone deacetylases promoting transcription of oncogenes including c-Myc and SRSF10 (Latham et al., 2012; San-Millán et al., 2019; Martinez-Outschoorn et al., 2011; Pandkar et al., 2023); 3) acetyl CoA promotes histone acetylation and lipogenesis, with the second being a key promoter of malignant transformation (Messier et al., 2016; Menendez and Lupu, 2007); 4) mutations in the TCA enzyme isocitrate dehydrogenase have been associated with depletion of a-ketoglutarate and increased production of 2-hydroxyglutarate (2-HG), which inhibits histone demethylation, while promoting HIF-1α activation (Liu et al., 2023; Lu et al., 2012; Turcan et al., 2012); 5) metabolic receptors not involved in energy metabolism, such as the aryl hydrocarbon receptor (AhR) have been shown to dictate epigenetic reprogramming in breast cancer: AhR has been linked to gene regulation of oncogenes (e.g., *BRCA1*) through hypermethylation of CpG islands, increased three-methylation and deacetylation of H3K9, increased levels of DNA methyltransferases 1, 3a and 3b, as well as higher levels of methyl binding protein 2 (Thakur et al., 2022; Hockings et al., 2006; Papoutsis et al., 2012).

Thus, tumour metabolism, oxygen deprivation and hormone signalling are interlinked processes that significantly impact the epigenetic landscape of breast cancer, modulating oncogenesis, survival, proliferation and metastasis (Figure 1). This highlights the importance of a better understanding of the co-dependence between hypoxia and metabolic reprogramming in breast cancer, as a tool for changing the epigenetic landscape and creating novel therapeutic approaches.

### Hypoxia and immune evasion

Generally, breast cancer is notorious for its low response to immune-targeted therapies, due to low tumour immunogenicity (Savas et al., 2016; Debien et al., 2023). However, the degree of immune infiltration is considered a good prognostic factor for TNBC patients (Savas et al., 2016; Dieci et al., 2021). The hypoxic microenvironment associated with breast tumours often impacts the physiology of immune cells, alongside that of the malignant tumour cells. As such, in infiltrating macrophages, the activity of histone demethylases is decreased, therefore increased expression of demethylases JMJD1A, JMJD2B, and JMJD2D is observed as a compensatory mechanism for lack of function. Thus, despite increased demethylase expression, H3K9 and H3K36 methylation is upregulated (Tausendschön et al., 2011). Such elevated methylation patterns decrease the ability of macrophages to produce immune cell-recruitment chemokines such as Monocyte chemoattractant protein-1 (MCP1), CC-type chemokine receptor 1 (CCR1) and CC chemokine receptor type 5 (CCR5), all of which regulate the migration of macrophages, facilitating tumour immune infiltration (Tausendschön et al., 2011).

Hypoxia also impacts the expression and activity of a master immune-regulator transcription factor, NF-kB. Although the mechanism through which low oxygenation activates NF-kB is not fully understood, several papers have proposed possible mechanisms (Cummins et al., 2006; Culver et al., 2010; D’Ignazio and Rocha, 2016). One such mechanism states that hypoxia facilitates the release of NF-kB from its inhibitory complex with the Inhibitor of IkB protein (IKK), by direct interaction of the latter with PHD1 proteins, otherwise complexed with HIF-1α under normoxia (Cummins et al., 2006). Another possible mechanism involves hypoxia induced activation of the Ca^2+^/calmodulin response, which further activates the Transforming Growth Factor Activating Kinase 1 (TAK1), leading to activation of IKK, and subsequent nuclear translocation of NF-kB (Culver et al., 2010). Under low oxygen conditions, NF-kB also plays a role in regulating the tumour-immune response. As such, hypoxia-induced NF-kB was shown to differentially regulate the expression of two chemoattractants: IL-8 (neutrophil recruiter and angiogenic factor) and MCP1 (recruiter of macrophages, monocytes, Natural Killer (NK) cells and lymphocytes). While MCP1 was suppressed by NF-kB via deacetylation of histones at the MCP1 promoter, IL-8 was unregulated, despite structural similarities between the two promoters (Safronova et al., 2009). Furthermore, NF-kB has been shown to enhance transcription of HIF-1α and β regulated genes (Rius et al., 2008; van Uden et al., 2011). As a result of both the increased expression of HIF-1α-regulated genes and increased IL-8 we see promotion of tumour formation and growth by enhancing vascularisation, as well as by regulating hormone receptor signalling (Waugh and Wilson, 2008).This fine tuning of gene expression via transcription factor activation and epigenetic remodelling, highlights the vast impact of hypoxia on cellular physiology.

Oxygen deprivation can also regulate tumour-immune evasion by activating the autophagy pathway. HIF-1α can function as an enhancer of Beclin1-VPS34 autophagolysosome formation, via activation of E1B-nineteen kilodalton interacting protein (BNIP3), thus promoting unfolded protein response (UPR) in cancer cells as well as in immune cells (Azad and Gibson, 2010; Monaci et al., 2022; Chipurupalli et al., 2019; Díaz-Bulnes et al., 2019). HIF-1α is also an activator of the protein tyrosine phosphatase PTP-PEST, which is an upstream activator of AMPK signalling potentiating autophagic responses (Yong et al., 2022). Autophagy can be both beneficial and detrimental for cancer cells. On the one hand, it can trigger degradation of aberrant proteins and thus prevent the development of malignancies, and on the other hand it can facilitate survival under low nutrient conditions by promoting energy production through the degradation of self-structures. AMPK is known as a tumour suppressor, inhibiting the metabolic transformation that sustains oncogenesis, as well as functioning as an inhibitor of cyclooxygenase 2 (COX2) and downregulating cancer stemness (Keerthana et al., 2023). However, hypoxia-induced AMPK activation has been linked with malignancy in the literature; in ER^+^ and TNBC cells, AMPK activation has been shown to promote resistance to chemotherapy (e.g., Doxorubicin) (Keerthana et al., 2023; Yu et al., 2021; Pan Y. et al., 2017). AMPK activation has also been shown to contribute to tumorigenesis by interfering with the redox balance. As such, AMPK phosphorylates and thus inactivates acetyl CoA carboxylase, preventing NADPH usage for fatty acid synthesis, and in turn producing more NADPH via fatty acid oxidation, therefore sustaining the energetical needs of the cell (Jeon et al., 2012). In some cancers, AMPK activation also leads to accumulation of acetyl CoA, which serves as a substrate for histone acetylases, thus promoting epigenetic reprogramming (Jiang et al., 2019); alternatively AMPK was also shown to directly phosphorylate histone 2B at serine 36, thus inducing epigenetic regulation of cellular metabolism (Bungard et al., 2010). In immune cells, AMPK plays a crucial role for survival. In T cells, AMPK activation facilitates memory cell formation by inhibiting mTORC1, as well as facilitating survival and activity of effector T cells under low glucose conditions, by promoting glutamine-based oxidative phosphorylation (Yang and Chi, 2015; Rolf et al., 2013). Furthermore, AMPK might play a role in macrophage polarisation by interfering with NAD^+^ acetylation and suppressing HIF-1α and NF-kB (Kim, 2018). In the context of tumour-immune interaction AMPK has been shown to have immune checkpoint inhibitory activity, which means that AMPK interferes with the expression/activity of cellular markers that function as immune cell inhibitors and promote cancer progression. Thus, AMPK has been shown to downregulate the immune checkpoint protein Programed Death-1 (PD-1) by interfering with p38 signalling as part of the mevalonate pathway, as well as to act synergistically with anti-PD-L1/Cytotoxic T-Lymphocyte Antigen-4 (CTLA-4) towards tumour clearance (Afzal et al., 2018; Pokhrel et al., 2021). Interestingly, decreased tumour hypoxia was also associated with a better response to anti-PD-1 therapy, which suggests that physiological AMPK and stress/hypoxia induced AMPK overactivation might have conflicting effects on the solid tumour microenvironment (Zandberg et al., 2021).

Glucose metabolism is also linked to immune regulation of the tumour microenvironment. In tumour cells glucose-based energy production is regulated by epigenetic mediators, such as long non-coding RNAs (lncRNAs), either directly by controlling the levels of glycolytic enzymes or glucose transporters (e.g., hexokinase 2, lactate dehydrogenase A (LDHA), pyruvate kinase isoenzyme M2, and pyruvate dehydrogenase kinase 1), or indirectly by interfering with signalling pathways such as PI3K/PKB, AKT/mTOR and Wnt/Snail (Lin, 2020; Tan et al., 2021). In tumour infiltrating T lymphocytes, low levels of glucose trigger binding of Glyceraldehyde-3-phosphate dehydrogenase (GAPDH) to IFNγ transcripts, this interferes with histone acetylation preventing differentiation of CD4^+^ T cells into the immunogenic Th1 phenotype (Chang et al., 2013). In TNBC, IFNγ stimulation is particularly important, as it enhances the expression of the immune checkpoint and tryptophan catabolic enzyme indoleamine 2,3-dioxygenase 1 (IDO1) by breast cancer cells. IDO1 then functions as a nutrient scavenger, starving the effector T cells from the tumour microenvironment of tryptophan, as well as inducing differentiation of tumour protective T cells (Treg) via its immunosuppressive reaction product, kynurenine (Sarangi, 2023). However, in ERα^+^ breast cancer, the IDO1 promoter is silenced through hypermethylation, thus suggesting that ERα expression on breast cancer cells might impact their response to microenvironmental stimuli through epigenetic reprogramming (Dieci et al., 2021). Furthermore, low glucose provides a survival advantage for Tregs, due to their reliance on fatty acid oxidation and increased AMPK activity, as well as due to FOXP3-mediated suppression of glycolysis, thus promoting tumour immune evasion (Abou Khouzam et al., 2022; Angelin et al., 2017; Gualdoni et al., 2016; Wu Q. et al., 2021). High lactate and low pH inhibit NK cytotoxicity and cytokine production, and dendritic cell (DC) maturation, but enhance naïve T cell differentiation to Tregs and infiltration of T, NK and myeloid-derived suppressor cells, leading to tumour progression (Abou Khouzam et al., 2022).

Taken together, these observations provide a more ample perspective on the epigenetic impact of tumour hypoxia, highlighting the need to understand the tumour-immune communication in order to develop more effective therapies for breast cancer patients.

### Hypoxia and oncogenes

The *BRCA1/2* genes are well-studied tumour suppressors, and mutations in these genes have been shown to promote oncogenesis for breast cancer in particular, but have also been associated with ovarian cancer and to a lesser extent prostate and endometrial cancers (Werner, 2022). *BRCA1* mutations are associated with 20%–25% of breast cancers (Mehrgou and Akouchekian, 2016). Hypoxia can modulate breast cancer progression by inhibiting methylation of H3K4, increasing H3K9 methylation and decreasing H3K9 acetylation in the promoter of the *BRCA1* gene. These modifications then drive the silencing of the *BRCA1* tumour suppressor gene, which could be reversed by treatment with histone deacetylase inhibitors, but not by DNA methylation inhibitors (Lu et al., 2011). Interestingly, the same study shows that hypoxia had a similar effect on the promoter of the *RAD51* gene. The RAD51 protein is known to interact with a series of proteins involved in breast cancer regulation including BRCA1/2 (Lose et al., 2006). Furthermore, under hypoxia, the *VEGF* promoter has been reported to bear opposite modifications to that of the *BRCA1* promoter, leading to enhanced the transcription of vascular growth factor proteins (Lu et al., 2011). Additionally, oxygen deprivation has been shown to upregulate a range of microRNAs associated with oncogenic potential in breast cancer, such as miR210, miR107 miR21, while upregulating miR20b, which in breast cancer is a VEGF inhibitor (Bao et al., 2012). Overall, while in breast cancer, hypoxia might exert a complex set of opposing interactions on VEGF, these findings demonstrate that hypoxia can epigenetically silence the key tumour suppressor BRCA1/2 increasing genomic instability with tumours.

### Summary

The complexity of the tumour microenvironment leads to an interconnected set of processes that are altered in the same direction to enhance the progression of the tumour. Hypoxia seems to be a master regulator of tumour oncogenesis, promoting metastatic behaviour via metabolic changes within the cancer cells which alter histone methylation and acetylation patterns; impacting on immune cell activation and impairing tumour clearance (Coronel-Hernández et al., 2021; Abou Khouzam et al., 2022; Camuzi et al., 2019). All these argue in favour of the need to better understand the intertwined networks controlled by oxygen deprivation, as well as justifying the need to develop novel therapies that could target different branches of the complex hypoxia-triggered signalosome with the ultimate aim of improving cancer-free survival in the clinic.

## Hypoxic regulation of the oestrogen receptor transcriptome in breast cancer

**FIGURE 2:**
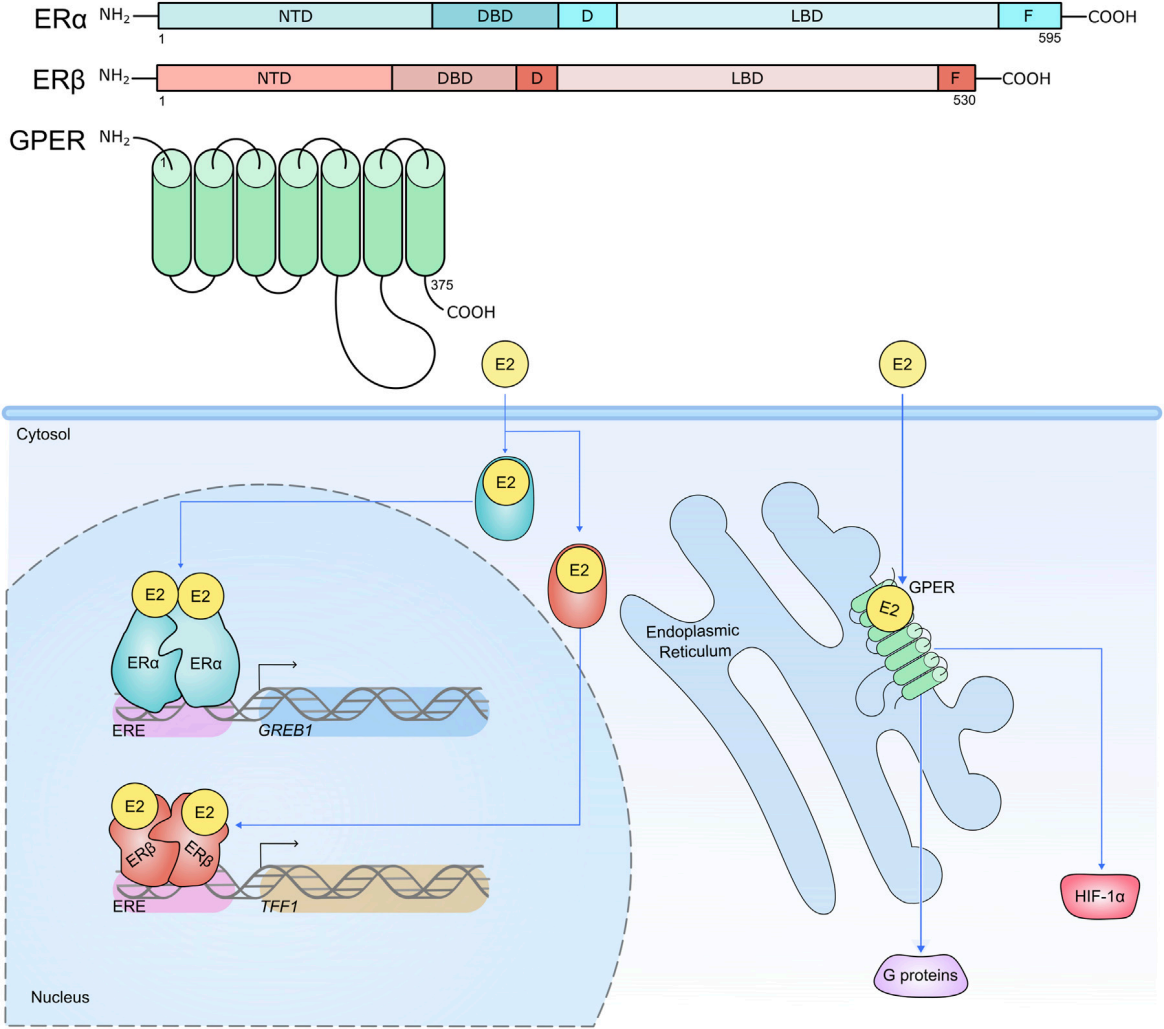
Structure of ERs, their cellular locations and activities. Nuclear receptors ERα and ERβ are compartmentalised into distinct functional regions. The N-terminal domain (NTD) houses activation function (AF) domain 1. The DNA binding domain (DBD) interacts with oestrogen response elements (EREs) proximal to target genes. The hinge region (D) contains a nuclear localisation signal and acts as a flexible region between the DBD and ligand binding domain (LBD). The LBD contains a hormone binding pocket where 17β-Estradiol (E2) can associate with the hormone receptors. The LBD is therefore important for the functional activity of the ERs in response to hormone stimulation. The F region is responsible for regulating gene transcription in a ligand-specific manner. ERα and ERβ reside in the cytosol awaiting stimulation by their steroid ligand E2, which induces a conformational change and subsequent translocation of the receptors to the nucleus where they are able to enact their transcriptional activities on target genes, predominantly through receptor binding to EREs (e.g. ERα binds to an ERE upstream of target gene *GREB1*; ERβ binds to an ERE upstream of target gene *TFF1*) (Klein-Hitpass et al., 1989; Wärnmark et al., 2003; Schwabe et al., 1993; Kumar et al., 2011). Conversely the GPER is expressed on the plasma membrane and intracellular membranes such as the endoplasmic reticulum, and contains seven transmembrane domains. Like ERα and ERβ, the GPER has high affinity for E2, and its role is to mediate the non-genomic effects of E2 within the cell, including mediating the activation of HIF-1α, and proteins involved in intracellular signalling cascades (Filardo et al., 2000; Filardo et al., 2002; Thomas et al., 2005; De Francesco et al., 2014; Vo et al., 2019).

Oestrogen receptors (ERs) are comprised of nuclear receptors (ERα and ERβ) and G-protein oestrogen receptors (GPERs), which orchestrate biologic effects in response to their steroid compounds (Walter et al., 1985; Kuiper et al., 1996; Carmeci et al., 1997) (Figure 2). In breast cancer, the ERα is the prolific driver of progression for approximately two-thirds of incidences (Harvey et al., 1999; Perou et al., 2000; Sørlie et al., 2001). While the presence of ERα promises a more favourable 5-year outcome compared to ERα-negative breast cancers, ERα-positive patients have a greater long-term risk of fatal disease as a consequence of *de novo* and acquired resistance to conventional anti-oestrogen therapy, and disease recurrence, even several decades after the primary diagnosis (Pan H. et al., 2017; Lindström et al., 2018). Hypoxia-mediated induction of HIF-1α has been strongly linked to breast cancers developing therapy resistance, which may present an interesting opportunity to explore therapeutics to target the HIF axis in endocrine-resistant disease (Yong et al., 2022; Gray et al., 1953; Samanta et al., 2014). In response to the presence of oestrogen or alternative ligands, ERα functions by the recruitment of a large scale protein complex (Papachristou et al., 2018; Holding et al., 2019) formed of a wide range of epigenetic components, including histone acetyltransferases and chromatin remodelers to drive gene expression through altering the epigenetic landscape and via the mediator complex. Here, we explore hypoxia-HIF-ERα signalling in breast cancer progression.

### Hypoxia, oxidative stress and ERα activity

Expression of hypoxia markers HIF-1α and carbonic anhydrase IX (*CAIX*) have significant prognostic implications in ERα positive breast cancer, where their increased abundance correlates with decreased disease-free survival in patients with luminal A or luminal B disease. Conversely, HIF-2α is not linked to disease outcome (Shamis et al., 2022). Investigations into the effect of hypoxia on ERα stability have been conducted, as loss of ERα expression is one of the most important events in the development of therapy resistance in ERα positive disease (Lindström et al., 2018; Lindström et al., 2012). When MCF-7 and T-47D breast cancer cell lines were cultured in 1% O_2_ for 8–24 h, *ESR1* mRNA was significantly reduced as a consequence of transcriptional repression in a HIF-1α-dependent manner (Ryu et al., 2011). Repression of *ESR1* was breast cancer specific, as human endometrial Ishikawa cells, which are also positively regulated by oestrogens and the ERα, did not show hypoxia-mediated transcriptional inhibition (Ryu et al., 2011). Hypoxia-dependent ERα regulation is multifaceted. Prolonged culture of MCF-7 and T-47D cells in 1% O_2_ induces degradation of ERα and ERβ protein (Wolff et al., 2017). Furthermore, siRNA knockdown of HIF-1α in these hypoxic cell lines conferred a strong induction of ERβ protein. These findings suggest that HIF-1α is an important regulator of the ERα/ERβ ratio. It is of interest that in MDA-MB-231 TNBC cells, CoCl_2_ induction of stable HIF-1α increased ERα expression in the otherwise steroid receptor-deficient model. However, the induction of ERα may be due to unspecified effects of CoCl_2_ treatment and not as a consequence of HIF activity, as the same CoCl_2_ treatment of MCF-7 and T-47D cells did not confer ERα degradation observed following hypoxic incubation (Wolff et al., 2017). Additionally, a comprehensive investigation into ERα stability in a panel of 10 ERα positive breast cancer cell lines show hypoxia induces degradation of the steroid receptor in all models, regardless of basal ERα abundance, and this is dependent on functional proteasome and HIF-1α activity (Padró et al., 2017). In fact, investigation of primary luminal A tumour heterogeneity suggests the expression of the ERα is negatively correlated with the expression of HIF-1α within a cycling hypoxia microenvironment (Kang and Li, 2022). Perhaps surprisingly, a physical interaction between ERα and HIF-1α has been observed by co-immunoprecipitation in MCF-7 cells transfected with HIF-1α/VP16 hybrid molecules. This study demonstrated that the physical interaction between HIF-1α and the ERα was important for the induction of proteasomal degradation of the steroid receptor (Cho et al., 2005). It is plausible therefore, that a level of cooperation and negative-regulation between the two transcription factors may exist (Yang et al., 2015). The converse is also true, demonstrating that ERα and HIF-1α are tightly connected, with ERα knock-down in ER^+^ models able to compromise the function of HIF-1α under hypoxic conditions (Yang et al., 2015). Some controversy surrounds the suitability of cell lines as a model for disease response to applied environmental stimulus (Gillet et al., 2013). Indeed, while standard 2D culture of breast cancer cell lines suggests the ERα is negatively regulated by O_2_ limitation, 3D culturing of T-47D breast cancer cell lines maintains ERα protein levels. In both models, ERα transcriptional activity is impaired following culture in hypoxia (Whitman et al., 2019). Nevertheless, the link between HIF-1α expression and poor disease-free survival for ERα positive breast cancer patients is apparent, and should be further investigated.

Oxidative stress in ERα positive breast cancer is indicative of aggressive disease, whereby genes responsive to both ERα and oxidative stress are linked to cancer cell growth and invasion pathways (Yau and Benz, 2008). Oxidants have been shown to impair ERα transcriptional activity by attacking zinc finger cysteine residues within the steroid receptor’s DNA binding domain, which in turn impedes ERα dimerisation and direct DNA binding (Whittal et al., 2000; Atsriku et al., 2005). There is evidence that the activity and expression of the ERα is regulated through epigenetic modification, which can be altered in response to O_2_ and reactive oxygen species (ROS) availability. Hypoxia increases the abundance of ROS by acting on complexes I, II, and III of the mitochondrial electron transport chain (ECT). Additionally, increased ROS production destabilises PHD enzymes, which further enhances HIF stabilisation (Wang et al., 2007; Kondoh et al., 2013). MCF-7 cells exposed to hydrogen peroxide (H_2_O_2_) as a source of ROS for extensive periods of time have increased cell growth, colony formation and upregulation of pro-metastatic genes such as *VEGF* and *WNT1,* conferring a more aggressive phenotype (Mahalingaiah and Singh, 2014). Furthermore, MCF-7 cells adapted to chronic oxidative stress are significantly less responsive to 17β-Estradiol stimulation and Tamoxifen antagonism, with demethylating agent 5-Aza-2′-deoxycytidine (5-aza-dC) re-sensitising cancer cells to the oestrogen and anti-oestrogen agents (Mahalingaiah et al., 2015). The reduction in responsiveness to ERα-targeting compounds was concurrent with increased expression of DNA methylating enzymes associated with gene silencing, *DNMT1* and *MBD4,* and decreased expression of *ESR1* mRNA and ERα protein. Incubation with 5-aza-dC was able to reverse these alterations in ERα expression, suggesting the mechanism of ROS-induced ERα instability was in part due to gene-silencing by the DNA methylating activities of *DNMT1* and *MBD4* (Mahalingaiah et al., 2015). DNA methylation of *ESR1* has been described in ERα negative MDA-MB-231, MDA-MB-468 and Hs578T cells (Ottaviano et al., 1994). Importantly, methylated ERα has been observed in clinical specimens (Yoshida et al., 2000). Indeed, a study found that in 41% of primary breast cancer tumours investigated, the promoter of the *ESR1* gene was hypermethylated, which strongly correlated with reduced ERα protein expression and more advanced disease (Ramos et al., 2010; Wei et al., 2012). The activity of histone deacetylases (HDACs) are also important in breast cancer progression, by altering the chromatin condensation and acetylation signature surrounding important oncogenes. Thus, HDAC inhibitors such as trichostatin (TSA) have been investigated for potential therapeutic benefit in aggressive breast cancer. In hypoxic MCF-7 cells, concurrent treatment with 100–300 nM TSA further enhanced hypoxia-mediated proteasomal degradation of the ERα. Conversely, in TNBC cell lines MDA-MB-231 and MDA-MB-435, incubation with TSA induces expression of the ERα (Yang et al., 2000; Yang et al., 2001; Bovenzi and Momparler, 2001) and enhances activity of ERβ, sensitising TNBC cells to anti-oestrogens such as tamoxifen (Jang et al., 2004). Further studies have implicated epigenetic modification in the silencing of the *ESR1* gene. In particular, the basic helix-loop-helix transcription factor TWIST has been shown to downregulate ERα expression by (i) recruiting DNA methyltransferase 3B (DNMT3B) to ERα′s promoter which induces methylation and (ii) re-organising the structure of the chromatin around ERα promoter by assembling HDAC1 (Vesuna et al., 2012). Hypoxia enhances the expression of TWIST through HIFα activity, and drives EMT in solid tumours (Yang MH. et al., 2008; Sun et al., 2009; Zhang L. et al., 2013).

Furthermore, oestradiol can impact on the redox balance in breast cancer via GPERs and non-canonical nuclear receptors such as ERα36 (Hall and Filardo, 2023; Ishii and Warabi, 2019; Thiebaut et al., 2020). The latter is an alternative promoter variant of the ERα, encoded by the *ESR1* gene, which has been associated with poor prognosis of mammary cancers and resistance to oestrogen therapies (Thiebaut et al., 2020). ERα36 and GPERs can mediate breast cancer progression via non-genomic mitogenic oestrogen signalling and ultimate activation of the Nuclear factor erythroid 2-Related Factor 2 (NRF2) (Ishii and Warabi, 2019; Xu F. et al., 2023). Stimulation of ERα36 and GPERs via 17β-oestradiol induces synthesis of superoxide radicals, through Src-induced activation of NADPH Oxidase 1 (NOX1); superoxyde then enhances ceramide production by nSMase2; next, ceramide functions as a single mediator which activates downstream cascades for casein kinase 2 (CK2) and EGFR signalling, both of which contribute to an increase in NFR2 activity/levels by stabilising it, promoting its nuclear translocation and inhibition of its degradation (Ishii and Warabi, 2019). NRF2 then regulates expression of genes involved in maintaining the cellular redox balance, such as members of the glutathione synthesis pathway (Ishii and Warabi, 2019; Ishii et al., 2000). The link between oestradiol signalling, GPER and NRF2 has been recognised beyond breast cancer, with the two proteins being interlinked and contributing to attenuated symptoms in atherosclerosis or preventing ferroptotic cell death in lung cancer cell lines (Feng et al., 2021). Oestradiol further upregulates levels of glycolytic intermediates as well as those of enzymes belonging to the pentose phosphate pathway, such as glucose-6-phosphate dehydrogenase (G6PD), potentially linked to mTORC1 over-activation; mTORC1 then promotes anaerobic glycolysis, cancer growth and metastasis (Sun et al., 2014). Interestingly, G6PD was shown to be essential for NRF2 function, as depletion of G2PD impairs survival of cells that constitutively express NRF2, and this effect can be reverse by supplementation with precursors of tricarboxylic acid (TCA) cycle metabolites (Ding et al., 2021). This mechanism shows the complex interplay of NRF2 not only in regulating gene expression but also in controlling the metabolic and redox balance in cancer. Under hypoxia, NRF2 is stabilised by HIF-1α-induced miR-101, while HIF-1α itself is a downstream target of NRF2 regulation (Thakur et al., 2022; Guo et al., 2015; Bi et al., 2021).

### Hypoxia, HIF-1α and anti-oestrogen therapy resistance

Hypoxia has been established as a key driver in the acquisition of therapy resistance, reviewed in (Chen et al., 2023; Muz et al., 2015). In the context of ERα positive breast cancer, several studies have investigated how hypoxia initiates this fundamental element of disease progression (Jögi et al., 2019; Yang et al., 2015).

A key event in the development of endocrine resistance is the loss of a functional ERα, either through degradation, truncation or posttranslational modification (Musgrove and Sutherland, 2009). As described above, hypoxia-mediated epigenetic modification of the *ESR1* gene has significant implications on the expression of the ERα, and desensitisation of breast cancer cells to anti-oestrogen therapy through *ESR1* gene silencing. Additional mechanisms of endocrine resistance through epigenetic alterations and the activity of HIFs have been identified.

Hypoxic MCF-7, CAMA-1 and T-47D breast cancer cell lines show decreased ERα expression and are less sensitive to tamoxifen and fulvestrant, compared to cells cultured in normoxia. Prolonged exposure (72 h) of breast cancer cell lines show stable HIF-2α protein accumulation, whereas HIF-1α was abundant after 6 h of hypoxia and declined at 72 h. HIF-2α also accumulated in normoxic endocrine-resistant cell lines, with consequently lower levels of ERα compared to normoxic hormone-sensitive parent cell lines. By inhibiting HIF-α with FM19G11 in the endocrine-resistant breast cancer cell lines, anti-oestrogen sensitivity can be restored. In fact, normoxic endocrine-resistant breast cancer cells showed significant decrease in cell viability when treated with FM19G11 and fulvestrant or tamoxifen (Alam et al., 2016). This study suggests that HIF-α accumulation is an important driver of endocrine resistance, even in the presence of oxygen, and may therefore serve as a beneficial therapeutic target in advanced Luminal A or Luminal B disease.

One of the most frequently mutated chromatin remodelling systems in cancer is the 15-protein-containing tumour suppressor complex SWI/SNF (Kadoch et al., 2013). In the SWI/SNF chromatin remodelling complex, a mutated AT-rich interaction domain 1A (*ARID1A)* subunit is enriched in metastatic ERα-positive breast cancers which are no longer responsive to endocrine therapy (Razavi et al., 2018; Xu et al., 2020). Here, persistent selective targeting of the ERα drives breast cancer progression towards a basal-like ERα-independent disease by depleting chromatin accessibility of ERα and pioneer factor forkhead box protein A1 (FOXA1), and reducing H3K27ac, a mark of active enhancers. Thus, anti-oestrogen therapies are no longer effective in preventing ERα-driven breast cancer progression (Xu et al., 2020). FOXA1 itself is an important chromatin remodeler that has a significant role in modulating the ERα transcriptome. In endocrine-resistant breast cancer, FOXA1 is significantly amplified and enhances tumour aggressiveness by activating IL-8 signalling to further promote tumour invasion, metastasis and therapy resistance (Britschgi et al., 2012; Singh et al., 2013; Fu et al., 2016). Additionally, FOXA1 transcriptional reprogramming increases the frequency of active enhancer H3K27ac and H3K4me1 marks deposited across the chromatin. Genes within the vicinity of the gained active enhancer marks are associated with pro-proliferation, anti-apoptosis and developmental signalling (Fu et al., 2019). Interestingly, HIF-2α observed increased FOXA1 binding and significant induction in a FOXA1-dependent, hypoxia-independent manner, which was associated with ERα-positive metastasis to the liver, pancreas and bone (Fu et al., 2019).

Monocarboxylate transporters (MCTs) are upregulated in cancers due to the increased glycolytic activity and higher lactate production. Inhibition of MCT4 results in significant lactate accumulation and HIF-1α protein levels. Upregulation of HIF-1α by either hypoxia or bandalit (MCT4 inhibitor) decreased ERα positive breast cancer cell line sensitivity to tamoxifen. Inhibition of HIF-1α resensitised the ERα positive breast cancer cell lines to tamoxifen (Nadai et al., 2021) Exploring cancer stem cell (CSC) activity using *in vitro* and *in vivo* models, including patient-derived tumour samples, Harrison et al. demonstrated HIF-1α-dependent CSC activity in hypoxic ERα positive cancers, a phenomena that was inhibited by blocking oestrogen and Notch signalling. Interestingly, CSC activity was decreased in hypoxic ERα negative cancers (Harrison et al., 2013). This may have significant implications when considering the selective blocking of hypoxia in different subtypes of breast cancer.

### Future directions for ER-targeted therapy development

Together, these findings suggest attenuation of HIF-1α or the proteasomal pathway may delay onset of endocrine resistance by maintaining stable ERα expression in hormone-driven breast cancers. Indeed, inhibition of the HIF-α axis in cancer development is of great interest, and many clinical trials have taken place to evaluate the efficacy of HIF attenuation in preventing disease progression (Wigerup et al., 2016). Re-introduction of the ERα in breast cancer by 5-aza 2′dC or TSA restores oestrogen responsiveness and sensitivity to current anti-oestrogen modalities, and provides additional therapeutic targets in the form of *DNMT1* or *HDACs* (Yang et al., 2001). FOXA1, which is a critical determinant of ERα transcriptome, is a key driver of endocrine resistance, independent of its role in modulating ERα gene targets (Fu et al., 2016; Fu et al., 2019). Therefore, attenuating the expression or activity of the pioneer factor may also be of therapeutic benefit. Interestingly, a proteolysis-targeting chimera (PROTAC) degrader of the SWI/SNF ATPase subunits has been investigated in prostate cancer models and ERα positive ZR-75-1 breast cancer cells, which showed FOXA1-driven cancer cells to be significantly sensitive to SWI/SNF attenuation (Xiao et al., 2022). Such therapeutic opportunities could be implemented in the treatment of FOXA1-driven, endocrine resistant breast cancer. It is likely that for advanced ERα breast cancer, a combination of therapies will be considered for the successful treatment of endocrine-resistant disease. For example, specific targeting of HIF-α with FM19G11 and future therapeutics in combination with fulvestrant or tamoxifen is able to restore therapeutic sensitivity in treatment-resistant breast cancer cell lines (Alam et al., 2016).

### Summary

**FIGURE 3:**
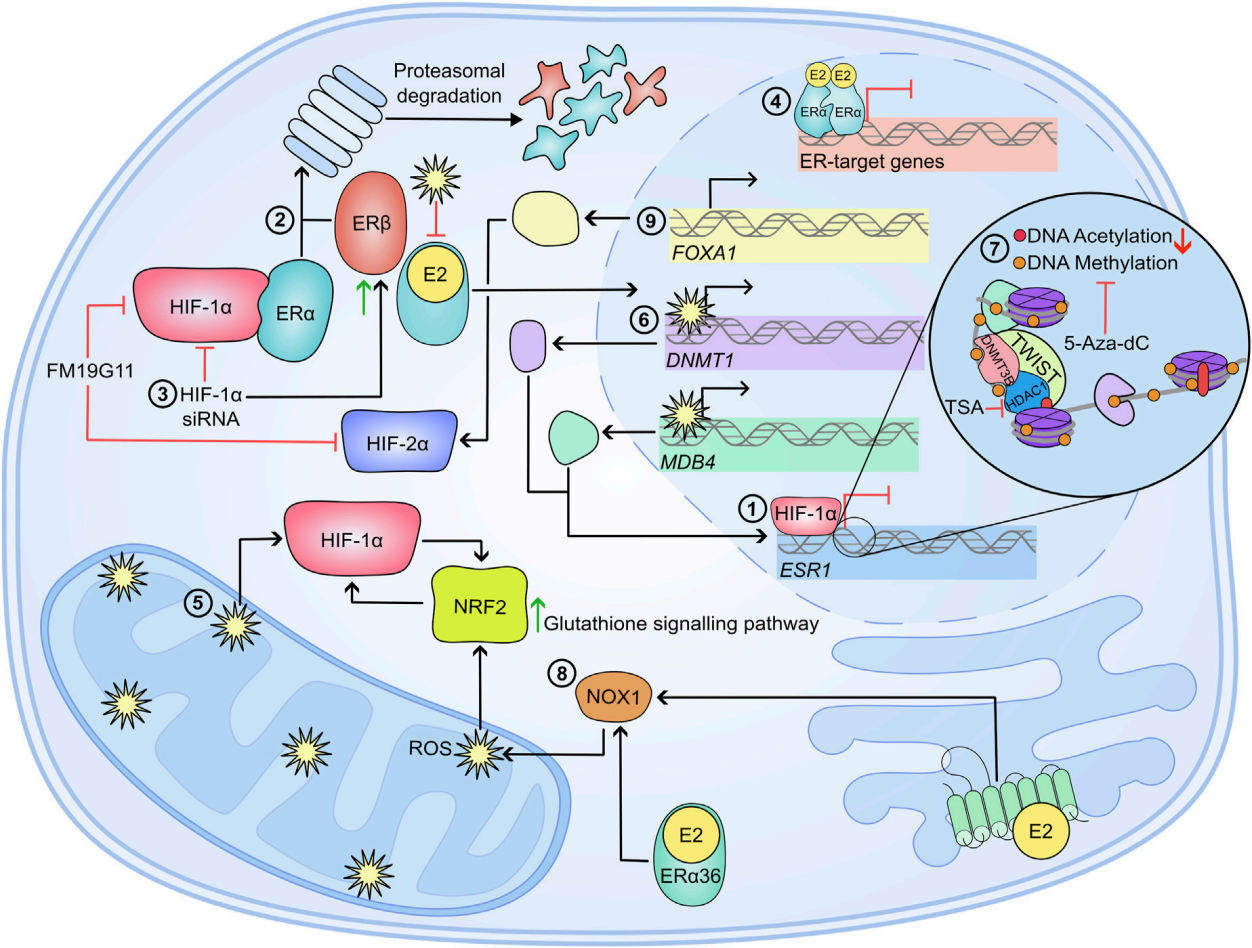
Mechanisms of therapy resistance in hypoxic breast cancer cells. In a hypoxic breast cancer cell 1) *ESR1* mRNA is significantly reduced in a HIF-1α-dependent manner (Ryu et al., 2011). 2) Hypoxia also causes proteasomal degradation of ERα and ERβ. For ERα, the proteasomal degradation is dependent on a physical interaction between the steroid receptor and HIF-1α. 3) Attenuation of HIF-1α via siRNA inhibits ERα knockdown, and enhances ERβ accumulation, suggesting HIFs are important in regulating the ERα/ERβ ratio (Wolff et al., 2017). 4) Alongside decreased ERα abundance, the transcriptional activity of the steroid receptor is impaired following prolonged culture in hypoxia (Whitman et al., 2019). 5) Reactive oxygen species (ROS) accumulate in hypoxia, which further enhances HIFα stability. ROS impedes ERα activity by preventing dimerization and DNA binding (Whittal et al., 2000; Atsriku et al., 2005; Wang et al., 2007; Kondoh et al., 2013). 6) Furthermore, ROS increases the expression of *DNMT1* and *MDB4* which may silence *ESR1* expression through their methylating activity. This *ESR1* silencing can be inhibited by 5-Aza-dC (Mahalingaiah et al., 2015). 7) Further epigenetic modifications silence *ESR1*; TWIST recruits DNMT3B and HDAC1 to induce hypermethylation of *ESR1* promoter and enhance a condensed chromatin structure (Vesuna et al., 2012). 8) Stimulation of ERα36 and GPER by E2 elicits a signalling cascade which ultimately induces NRF2 levels and activity, which further influences redox balance by inducing the glutathione signalling pathway (Ishii and Warabi, 2019). 9) FOXA1 is significantly amplified in endocrine resistant breast cancers, which can drive HIF-2α expression and enhance metastasis (Britschgi et al., 2012; Singh et al., 2013; Fu et al., 2016; Fu et al., 2019). All together, hypoxia and HIFα activity in ERα+ disease cause decreased sensitivity and effectiveness of anti-oestrogens such as Tamoxifen and Fulvestrant, which further drives disease progression, metastasis and correlates to poor disease outcome.

As summarised in Figure 3, in hypoxic breast cancer, ERα is degraded via the proteasomal pathway which is dependent on the interaction between HIF-1α and the ERα. Hypoxia also represses *ESR1* gene transcription through ROS accumulation and epigenetic reprogramming of *ESR1,* and histone modification. HDAC and methyltransferase inhibitors are able to restore functional ERα in breast cancer cells, and re-sensitise the cell lines to anti-oestrogen therapies such as tamoxifen and fulvestrant. Interestingly, estradiol stimulation via non-canonical ER receptors was also shown to contribute to maintaining the cellular redox balance via NRF2 signalling. Additionally, FOXA1 and the SWI/SNF chromatin remodelling complex may be beneficial therapeutic targets in advanced breast cancer. Overall, limited O_2_ availability and increased HIFα abundance has significant consequences for ERα+ disease by decreasing sensitivity to ER-targeting therapies such as fulvestrant and tamoxifen, disrupting the redox balance and enhancing tumour progression and metastasis.

## Hypoxia-driven stemness and chemoresistance in TNBC

### Hypoxia and breast cancer stemness

The self-renewal capacity of a subset of breast cancer cells is a particular feature of TNBC tumours (Fillmore and Kuperwasser, 2008; Gil-Gas et al., 2023) and is associated with a worse outcome for patients. Hypoxia contributes to this process by increasing this stem cell-like cellular compartment within tumours (Xie et al., 2016). In MDA-MB-231 cells, hypoxia shows a more significant effect on inhibiting apoptosis than cell proliferation, preventing the malignant cells from being cleared by immune cells in the microenvironment. Furthermore, hypoxia increases the proportion of the proliferative CD24^−^, CD44^+^, and Epithelial Specific Antigen (ESA)^+^ subtype of cells within the tumour, leading to a significantly higher colony formation rate (Xie et al., 2016).

HIF proteins typically coordinate the hypoxic response through modulating chromatin accessibility, through histone modifications, leading to gene expression changes (Stone et al., 2019; Terekhanova et al., 2023). However, when one of its target genes is being expressed, HIF proteins are able to increase expression more directly. RNA pol II is recruited to the promoter of HIF target genes during normoxia but transcription pauses shortly after beginning (Yang Y. et al., 2022). This pausing contributes to the regulation of 40%–70% of target genes whose expression is induced by physiological stimuli like heat shock and hypoxia (Bunch et al., 2014; Jonkers and Lis, 2015; Yamaguchi et al., 1999). Access to HIF target genes within these regions is aided by proteins like Chromodomain-helicase-DNA-binding protein 4 (CHD4); a protein that is increasingly implicated in promoting stemness and chemoresistance in breast cancer (Novillo et al., 2021; Zhang J. et al., 2022; D’Alesio et al., 2019; Wang Y. et al., 2020). Evidence suggests that both HIF-1α and 2α subunits are capable of physically interacting with CHD4, this interaction enhances the expression of HIF target genes, leading to increased proliferation of MDA-MB-231 cells (Wang Y. et al., 2020). Furthermore, it was found that CHD4 increased RNA pol II loading onto HIF target genes during normoxia via the histone acetyltransferase P300. Given the previously established regulation of HIF targets through stalling on RNA pol II (Yang Y. et al., 2022; Bunch et al., 2014), together this suggests CHD4 plays a large role in the regulation of HIF target gene expression. Enhanced HIF target expression is reinforced by data showing that loss of HIF expression can reverse the CHD4-mediated growth. Similarly, cells cultured in hypoxic conditions showed elevated CHD4 recruitment via HIFs and increased HIF recruitment, leading to further increased expression of HIF targets. In addition, HIF1α and 2α seem to be working in concert with each other over longer periods of hypoxia to maintain the cancer stem cell pool. HIF-1α activates *Nanog*, a pluripotency factor in embryonic cells that promotes cell survival, while HIF-2α promotes transcription of *POU5F1* and *Myc*, which drive self-renewal and cell proliferation respectively (Emami Nejad et al., 2021).

Indeed, in breast cancer HIF-1α and HIF-2α were shown to promote stemness by promoting expression of AlkB homolog 5 (ALKBH5), which promotes m^6^A demethylation of a cancer stemness promoter, the NANOG mRNA; this enhances NANOG expression and subsequently increases the breast cancer stem cell phenotype in TNBC cell lines (Zhang et al., 2016). Across breast cancer subtypes, including both TNBC and ER^+^ breast cancer, HIF- 1α and HIF- 2α expressions vary and overlap over time to coordinate a prolonged hypoxic response, known as the HIF switch (Jaśkiewicz et al., 2022; Serocki et al., 2018; Mutvei et al., 2018). Overexpression of HIF-2α activates Wnt and Notch signalling, inhibiting the wnt pathway with DKK-1 resulted in reducing stem-cell phenotype (Yan et al., 2018). Aberrant Notch signalling in breast cancer has been shown to coordinate the hypoxic response by upregulating HIF- 2α expression while down regulating HIF- 1α (Mutvei et al., 2018). Taken together, Notch signalling appears to work in tandem with HIF- 2α to orchestrate a long-term hypoxic response that enriches self-renewing cancer cells.

Circular RNAs (circRNA) form part of the human transcriptome that is increasingly implicated in promoting breast cancer stem cell transition (Zhan et al., 2021; Xu M. et al., 2023; Yang R. et al., 2022). CircHIF1A was identified in differential expression analysis of a circRNA array between hypoxic and normoxic CAF exosomes in breast cancer. Through sequestering miR-580-5p, CircHIF1A was shown to regulate CD44 which in turn enriched cell populations expressing OCT4, SOX2, ALDHA1, CD44, and Nanog (Zhan et al., 2021). Interestingly, although high circHIF1A exosomes in hypoxia stimulate stemness, the level of circHIF1A is lower during normoxia and maintains a smaller stem cell population. CircSTT3A is another circRNA that is upregulated significantly in hypoxia, via HIF-1α, and often leads to a poor prognosis (Xu M. et al., 2023). Through Hsp70, CircSTT3A stabilises PGK1, leading to increased serine synthesis, SAM and H3K4Me3 accumulation. The accumulation of H3K4Me3 results in the upregulation of stemness factors CD44, c-Myc and Klf4.

### Hypoxia and TNBC metastasis

The previously discussed long-term coordination of the hypoxic response through Notch signalling is concordant with data suggesting Notch signalling can stimulate the EMT transition in TNBC (Chen et al., 2010). Upregulation of several Notch target genes was observed during hypoxia, some of which, such as HES1, were bound by HIF-1α at the promoter. Hypoxia-driven gene expression changes resulted in decreased expression of E-cadherin, increased migration and invasion that could be reversed with the Inhibition of the Notch pathway (Chen et al., 2010). Notch and Wnt signalling pathways are key to the progression of many cancers, including breast cancer (Kumar et al., 2021; Braune et al., 2018). Twist is one of the regulatory proteins within the Wnt pathway with a hypoxic response element (HRE) within its promoter leading to its upregulation during hypoxia (Yang MH. et al., 2008). Similarly to Notch-driven changes, a metastatic phenotype was observed in HIF-1α overexpressing cells that could be abrogated through knockdown of TWIST with siRNA.

Long non-coding RNA (lncRNA) are also a key regulator during hypoxia that are increasingly implicated in breast cancer (Stone et al., 2019; Yang R. et al., 2022; Peng et al., 2021; Kuo et al., 2020; Maldonado and Melendez-Zajgla, 2022). The Wnt signalling axis is one such pathway that is reinforced through regulation by lncRNA RBM5 antisense RNA 1 (RBM5-AS1), a molecule found to be significantly upregulated in hypoxic breast cancer (Li et al., 2022b). RBM5-AS1 is upregulated by Runt-related transcription factor 2 (RUNX2) - which is also upregulated during hypoxia - with its overexpression driving proliferation and migration through the promotion of EMT. Several other lncRNA have been implicated in tumourigenesis within breast cancer patients, including NDRG1-OT1, which was first identified in MCF-7 cells (Chao et al., 2022; Lin et al., 2018). NDRG1-OT1 has since been shown to be directly regulated by HIF-1α during hypoxia as overexpression of HIF-1α resulted in upregulation which can be reversed with HIF-1α knockdown. Expression of NDRG1-OT1 promotes tumour growth and invasion, potentially by acting as a sponge for miR-875-3p, a microRNA (miRNA) found to suppress tumour proliferation and migration (Chao et al., 2022). Sponging of miRNAs appears to be a common trend amongst lncRNAs, Mitochondrial Oxygen-Responsive Transcript 1 (MTORT1), is an oxygen-responsive lncRNA that sequesters MicroRNA 26a-1 (miR-26a-5p) to regulate its target in the mitochondria of cells (Cheng et al., 2021). MTORT1 appears to play a tumour-suppressive role in cells as its knockdown leads to increased migration (Cheng et al., 2021). Sequestering miRNA increases breast cancer migration due to their regulatory role in key pathways during breast cancer progression. MicroRnma 18a (miRNA-18a) expression is suppressed in breast cancer cells that spontaneously colonise the lungs, likely through the direct activation of HIF-1α (Krutilina et al., 2014). Another lncRNA that was specifically identified in TNBC cells and was found to be specifically regulated by HIF-1α through binding at its promoter (Chen et al., 2021). lncRNA induced by hypoxia and abundant in TNBC (LnclHAT) was found to promote lung metastasis and cell proliferation through expression of nearby oncogenes pyruvate dehydrogenase kinase (PDK1) and Integrin Subunit Alpha 6 (ITGA6).

An investigation into the effects of chronic hypoxia (CH) and intermittent hypoxia (IH) on MDA-MB-231 found that although cell proliferation was significantly inhibited, expression of both HIF-1α and Vimentin was increased (Liu et al., 2017). However, a greater effect was observed in cells exposed to IH than in cells exposed to CH. Vimentin has been identified as a marker of the epithelial-mesenchymal transition (EMT) (Lei et al., 2015; Kallergi et al., 2011), indicating that IH could be a more potent driver of metastasis than CH. The effects of IH are highly relevant to breast cancer progression, as it often occurs when structural irregularities of the vasculature within the tumour impede the proper flow of blood, causing temporary shortages of oxygen within these regions of the tumour (Liu et al., 2010; Verduzco et al., 2015). IH was also found to significantly enhance the migratory ability and metastatic potential of cells exposed to it (Liu et al., 2017). The increased migration of these cells was then linked to the number of hypoxia-reoxygenation cycles the cells were exposed to and knockdown of HIF- 1α abolished this migration and increased Vimentin expression.

HIF proteins recruit epigenetic regulators to target genes to regulate the expression of those genes, however, these interactions can be shared by only one or by both isoforms of HIF. For example, JMJD2C is a histone demethylase that has been implicated in breast cancer progression during hypoxia but is specifically recruited to hypoxic response elements (HRE) by HIF-1α, not HIF-2α (Luo et al., 2012). Here, JMJD2C removes H3K9me3 marks and enhances the binding of HIF-1α leading to enhanced expression of key genes required for breast cancer progression (PDK1) and metastasis to the lung (LOXL2 and L1CAM). The expression of this gene in human tumours is strongly associated with JMJD2C expression. In mice where cancer cells have been injected into fat pads, the knockdown of JMJD2C inhibits the growth of cancer cells and spontaneous metastases to the lung (Luo et al., 2012).

Utilisation of JMJD2C by HIF-1α exemplifies how HIF signalling can create a positive feedback loop that continuously enhances HIF signalling and compounds its malignant effects. The downstream expression of PDK1 by JMJD2C has several effects on the cell (Wei et al., 2023). PDK1 is highly expressed in breast cancer tissues and its knockdown results in impaired growth and motility of breast cancer cells, in addition to inhibiting HIF-1α signalling. PDK1’s primary function is to inhibit its target protein, the pyruvate dehydrogenase complex, to switch the metabolism of the cell from the TCA cycle to glycolysis, one of the hallmarks of cancer. PDK1 is also capable of stabilising HIF-1α through phosphorylation at several serine sites, although serine 451 contributed most to this effect by preventing interaction with PHD proteins (Wei et al., 2023). The relationship between the two proteins goes even further as PDK1 can promote interactions with HIF and P300, enhancing the activity of HIF-1α and creating a positive feedback loop to promote breast cancer progression.

ZMYND8 is a transcription factor upregulated by HIF-1α and HIF-2α in human breast tumours that is correlated with poor outcomes for patients with high expression (Chen et al., 2018). Oncogenicity of ZMYND8 requires both HIF-1α and HIF-2α, allowing the trio to exert global influence of target genes through recruitment of bromodomain containing protein BRD4. Deletion of ZMYND8 reduces colony migration and formation, in addition to hampering lung colonisation in mouse models (Chen et al., 2018). The contribution of HIF- 2α towards metastasis in breast cancer can be further reinforced by the action of Peptidyl-prolyl cis-trans isomerase NIMA-interacting 1 (Pin1) (Guillen-Quispe et al., 2023). HIF-2α contains a pSer/Thr-Pro motif binding motif for Pin1 to bind during normoxia and hypoxia, prolonging the stability of HIF- 2α and enhancing migration.

### Hypoxia and chemoresistance

Paclitaxel (PTX) is a standard-of-care chemotherapy drug that is used to treat metastatic TNBC by targeting tubulin to disregulate spindle fibre formation and cell division (Wang et al., 2023; Hsu et al., 2021). However, resistance to the treatment can develop, leaving patients without sufficient treatment options (Han et al., 2021). Evidence in several TNBC and ER^+^ cell lines shows that treatment with PTX induces expression of both HIF-1α and HIF- 2α (Samanta et al., 2014; Yan et al., 2018). Expression of both HIF proteins goes on to upregulate IL-8 and IL-6 expression, which has previously been shown to decrease the survival of patients through enrichment of stem cell-like phenotypes (Marotta et al., 2011). Although in this case, the majority of the hypoxia-driven stem cell enrichment required HIF-1α, both proteins contributed to enrichment during normoxia after PTX-induced expression (Samanta et al., 2014). A further protein upregulated by HIF-1α implicated in PTX resistance is complement 1q binding protein (C1QBP) (Wu H. et al., 2021). C1QBP is often overexpressed in TNBC where, through decreased protein kinase C-nuclear factor-kappa B (PKC-NF-κB) during hypoxia, VCAM-1 was arrested, leading to increased invasion and PTX resistance. Inhibition of C1QBP reduced cell invasion and increased sensitivity to PTX as well as leading to downregulation of Multidrug Resistance 1 (MDR1). MDR1 encodes a membrane embedded efflux exporter protein, P-glycoprotein, that is directly responsible for resistance to many chemotherapeutic drugs (Wu H. et al., 2021; Schöning et al., 2017; Wang C. et al., 2020). Interestingly, HIF-2α coordinates alternative chemoresistance mechanisms over longer periods of time due to the previously discussed HIF switch (Jaśkiewicz et al., 2022; Serocki et al., 2018; Mutvei et al., 2018). Overexpression of HIF-2α activates Wnt and Notch signalling, inhibition of this Wnt signalling with DKK-1 is capable of reversing PTX resistance (Yan et al., 2018).

In TNBC lines as well as in xenograft models, HIF-1α was shown to induce resistance to another member of the taxane family, docetaxel, by downregulating the levels of mRNA494, which in turn leads to increased expression of Survivin (Li H. et al., 2022). Survivin belongs to the Inhibitor-of-Apoptosis Protein (IAP) class, and has been previously associated with increased chemotherapy and radiotherapy resistance in the majority of human cancer, including breast malignancies (Li H. et al., 2022; Grdina et al., 2013; Peery et al., 2017). Additionally, hypoxia can regulate drug resistance by altering the levels of cellular microRNAs, such as miR15b and miR-16, which have been shown to contribute to resistance to doxorubicin, vincristine, etoposide and cisplatin (Liao et al., 2014).

HIFs have also been associated with enhanced chemoresistance of cancer cells, under hypoxic conditions, upregulating other ABC transporters and thus promoting resistance to chemotherapy (Sun et al., 2009). HIF-1α in particular was shown to play an essential role upregulating the activity of two ABC transporters, MDR1 and BCRP (Schöning et al., 2017). Furthermore, hypoxia has also been shown to contribute to cancer resistance to radiotherapy; studies show that the hypoxic tumour microenvironment might induce stromal cell production of exosomes loaded with 5′-triphosphate RNA, this works as a simultaneous activator of RIG-1 and Notch3 signalling in breast cancer cells, which facilitates differentiation of radiation-resistant cancer cells (Boelens et al., 2014; Gilreath et al., 2020).

Hypoxia can also regulate immunotherapy response. Due to the lack of consistent cell markers, the high mutational burden and the high expression of PD-L1, some metastatic TNBC patients are suitable candidates to receive anti-PD-1 immunotherapy. However, resistance often arises in patients who receive the treatment (Chen et al., 2022; Ma et al., 2022). During hypoxia, immune effector genes of cells within the TNBC and HER2^+^ stroma are downregulated, resulting in a lack of tumour-infiltrating lymphocytes (TILs) (Ma et al., 2022). HIF-1α coordinates epigenetic changes by forming distinct regulatory complexes with HDAC1 and SUZ12, creating a dynamic equilibrium of histone acetylation and methylation. Therefore, HIF- 1α can coordinate the downregulation of immune effector genes, resulting in impaired cytotoxicity and resistance to anti-PD-1 blockade treatment (Ma et al., 2022). Inhibiting either HDAC1 or EZH2 was able to rescue the cytotoxicity of TILs and restore sensitivity to anti-PD-1 blockage treatment, however, the combined treatment showed the largest reduction.

Thus, hypoxia plays a complex role in mediating survival and resistance to a range of therapeutic approaches, which highlights the need for a better understanding of the mechanisms underlying such changes in breast cancer, as well as identifying hypoxia as an excellent target for novel therapeutics against breast cancer.

### Summary

Within TNBC, stemness and chemoresistance driven by hypoxia are often inextricably linked (Dave et al., 2012; Ervin et al., 2022; Gooding and Schiemann, 2020; De Angelis et al., 2019). Tumours can prepare for hypoxia by loading HIF target genes with paused RNA pol II, through recruitment of epigenetic factor CHD4 via p300 (Jonkers and Lis, 2015; Yamaguchi et al., 1999; Wang Y. et al., 2020). When oxygen levels drop, the RNA polymerase unpauses and stimulates the enrichment of stem-like cell compartments through increased homeobox protein production, such as NANOG via the Wnt and Notch signalling axes (Zhang et al., 2016; Mutvei et al., 2018; Chen et al., 2010; Kumar et al., 2021; Braune et al., 2018; Li et al., 2022b). Stimulation of pathways responsible for the development of the breast, in concert with non-coding RNAs upregulated by hypoxia results in expression of pro-self-renewal markers like OCT4 and CD44 in addition to strong drivers of cell proliferation such as Myc (Emami Nejad et al., 2021; Zhan et al., 2021; Xu M. et al., 2023; Yang R. et al., 2022). The enhanced survivability of these cells is further compounded by the gain of resistance to several of the limited therapeutic options available to patients. One of the mechanisms utilised by TNBC cells during hypoxia to induce chemoresistance is through altering the accessibility of key genes through control of histone markers via EZH2 and HDAC1 (Ma et al., 2022). Metastatic behaviour can be sustained and reinforced through epigenetic alterations catalysed by proteins such as JMJD2C in order to sustain expression of the required proteins to facilitate invasion into specific tissues - like LOXL2 and L1CAM aiding invasion to the lung (Luo et al., 2012). Taken together, both HIF-1α and HIF-2α are utilised in TNBC to enact large scale epigenomic alterations that enrich self-renewing cells, grant chemoresistance and promote migration and invasion to distant sites.

## Other hypoxic-induced epigenetic regulators in tumours

Maintaining the ionic balance between the intracellular and the extracellular compartments is essential for maintaining cellular physiology. In cancer, the dynamics of ions across the plasma membrane change to facilitate migration and metastasis (Webb et al., 2011; Monteith et al., 2017; Pardo and Stühmer, 2014; Ouwerkerk et al., 2003). Solid tumours are known to have elevated Na^+^ concentrations compared to healthy tissues. This observation was first recorded in glioma allograft tumours using flame photometry and X-ray microanalysis (Hürter et al., 1982). A number of studies have thereafter highlighted a link between hypoxia and Na^+^ dynamics in promoting breast cancer cell progression (Dewadas et al., 2019; Carroll et al., 2022; Takatani-Nakase et al., 2022; Liskova et al., 2019). Survival and migration of triple negative breast cancer cell lines, as well as their expression of the voltage-gated sodium channel Na_v_1.5 has been linked with expression of the α subunit of HIF1 (HIF-1α), with both HIF-1α and Na_v_1.5 being upregulated in the more aggressive TNBC cell lines, compared to other breast tumour subtypes (Dewadas et al., 2019). Hypoxic tumour regions are often associated with acidosis, and studies have shown that hypoxia can induce expression of Na^+^-dependent bicarbonate transporters, such as SLC4A4 and SLC4A5, which have been shown to promote EMT, hypoxic signalling and metastasis *in vivo* (Carroll et al., 2022). Another metastatic regulator of hypoxic tumours via pH modulation, is the type 1 Sodium Calcium Exchanger (NCX), which seems to form a complex with carbonic anhydrase IX and the NHE1, increasing proton export and thus causing acidosis (Liskova et al., 2019). Furthermore, in triple negative cell lines, hypoxia-induced invadopodia formation and subsequent migration and metastasis were also associated with NHE1 activation (Takatani-Nakase et al., 2022).

Alongside Na^+^, Ca^2+^ ions also play a key role in breast cancer progression. While Na^+^ and Ca^2+^ are intrinsically linked through the NCX, Ca^2+^ is also a major signalling molecule regulating a range of cellular responses, from G-coupled protein receptor signalling, to metabolism, gene expression and responses to cytokine mediated immune checkpoint regulation (e.g., IFNγ signalling inducing IDO1/PD-L1 expression) (James et al., 2023; Bagur and Hajnóczky, 2017; Nair et al., 2002). Apart from the well documented role of Ca^2+^ as a signalling agent, studies have also investigated its role in epigenetic processes. Thus, direct histone modifications (e.g., H3 phosphorylation, increased histone acetylation at the promoter of prolactin) or elevations in epigenetic modifier proteins such as histone acetyl transferases (e.g., p300) have all been associated with increases in Ca^2+^ release (Harper et al., 2021; Whitlock et al., 1980; Nair et al., 2006). Changes in cytosolic Ca^2+^ are quickly detected by Ca^2+^ sensing proteins such as calmodulin, which further regulates the activity of a range of transcription factors such as CREB and NF-kB (Kornhauser et al., 2002; Zhu et al., 2011). Another Ca^2+^ sensor of particular importance for breast cancer is the HIF-1α-induced S100A10 protein, which forms a complex with the autophagy related annexin A2 protein, the histone chaperone SPT6 and the demethylase KDM6A, mediating H3K27me3 demethylation at the site of the pluripotency regulatory gene OCT4, and thus promoting breast cancer stemness (Lu et al., 2020). Furthermore, peptidylarginine deiminase 4 (PAD4) is a Ca^2+^ dependent enzyme that citrullinated histone H1, targeting pluripotency mediators such as the oxygen-sensitive *de novo* methylase in human mesenchymal stem cells (Dntm3) and the transcription regulator, Tripartite motif-containing 28 (Trim28) (Christophorou et al., 2014); PAD4 also citrullinated H4R3, mediating the p53 apoptotic response (Christophorou et al., 2014; Arita et al., 2004; Tanikawa et al., 2012). Furthermore, PAD4 was also shown to inhibit cell cycle arrest by interacting with p53 and subsequently relocating to the promoter of p21 where it regulates histone citrullination and decreases methylation of H3R17 (Li et al., 2008).

Although hypoxic regulation has primarily been investigated in terms of intracellular molecular adaptation, oxygen depletion often impacts the tumour as a system, rather than purely affecting single cell adaptations. Tumours are dynamic entities which rely heavily on communication between the different compartments of the microenvironment. One method of achieving synchronised adaptations towards tumour progression, is via paracrine signalling using tumour-secreted nanoparticles called exosomes, that mediate a range of processes such as stromal communication, immunosuppression and division (King et al., 2012). In ER^+^ (MCF7s), HER2^+^ (SKBR3s) and triple negative (MDA-MB-231s) breast cancer lines, exposure to hypoxia or activation of HIF-1α signalling were shown to enhance secretion of exosomes carrying elevated levels of miRNA-210, which is believed to contribute to tumour progression by stimulating DNA repair, chemotaxis and survival of endothelial cells, thus enhancing tumour vascularisation (King et al., 2012; Fasanaro et al., 2008; Crosby et al., 2009). Further studies have shown that hypoxic exposure of ER^+^ and triple negative breast cancer lines also increases the production of microvesicles (MVs), through HIF-1α induced expression of the RAB22A GTPase, which co-localises with budding vesicles. Exposure of naive TNBCs to MVs was shown to increase metastasis and focal adhesion. Furthermore, in primary tumours, elevated RAB22A was associated with poor disease prognosis (Wang et al., 2014).

### Summary

The ionic microenvironment contributes to tumour metastasis and negatively impacts on disease prognosis, undergoing significant changes under hypoxic conditions that lead to cellular acidification, chromatin remodelling, EMT and metastasis, particularly through changes in intracellular Na^+^ levels (Hürter et al., 1982; Dewadas et al., 2019; Takatani-Nakase et al., 2022). Upregulation of specific Ca^2+^ transporters impacts the gene expression profile of breast cancer cells primarily via Ca^2+^-regulated proteins (e.g., PADs) but also via calmodulin activation and recruitment of transcription factors and related epigenetic components of the transcriptional complex, promoting cancer survival and stemness (Kornhauser et al., 2002; Zhu et al., 2011; Christophorou et al., 2014; Arita et al., 2004; Tanikawa et al., 2012). Furthermore, hypoxia was shown to expand its epigenetic impact also via paracrine signalling either via exosomes or EVs, contributing to enhanced metastasis and negatively impacting patient survival (Wang et al., 2014), (King et al., 2012). These observations highlight the pleiotropic effect of hypoxia on the tumour microenvironment, as well as highlighting potential therapeutic routes that require further exploration.

### Concluding remarks

These observations taken together reveal the heterogeneity of breast cancers and show the vast epigenetic implications of hypoxic exposure which impact on metabolic changes, hormone signalling, microRNA-dependent cancer stemness reprogramming and changes in ionic dynamics, highlighting the need for further research on the topic of oxygen-depletion and epigenetic reprogramming, as well as presenting the significant therapeutic potential of targeting hypoxia in breast cancer.

## Abbreviations

TNBC, Triple Negative Breast Cancer; ER, Oestrogen Receptor; HIF, Hypoxia-Inducible Factor; EMT, Epithelial-Mesenchymal Transition; VGSC, Voltage-Gated Sodium Channel; HDAC, Histone Deacetylases; OXPHOS, Oxidative Phosphorylation; GPER, G Protein-coupled Oestrogen Receptor; TCA, Tricarboxylic Acid; JHDMs, Jumonji C Domain-containing Histone Demethylases; TET, Ten-Eleven Translocation; AHR, Aryl Hydrocarbon Receptor; UPR, Unfolded Protein Response; lncRNA, Long non-coding RNA.
